# Complete Genome Sequence of Variovorax paradoxus JBCE486, Which Alleviates the Salt Stress of the Endemic Plant Ulleung-Sanmaneul

**DOI:** 10.1128/mra.01027-22

**Published:** 2022-12-01

**Authors:** Swarnalee Dutta, Yong Hoon Lee

**Affiliations:** a Division of Biotechnology, Jeonbuk National University, Iksan-si, Jeollabuk-do, Republic of Korea; University of Arizona

## Abstract

Variovorax paradoxus strain JBCE486, which was isolated from the rhizosphere of the endemic plant Ulleung-sanmaneul in South Korea, promoted growth and stress resistance in the common chive, as well as the model plant Arabidopsis thaliana, under salt stress conditions. Here, we report the genome sequence of JBCE486.

## ANNOUNCEMENT

Variovorax paradoxus is a Gram-negative, motile, rod-shaped bacterium that interacts with a variety of bacteria and plants. V. paradoxus is widely distributed in soil and water, with metabolically diverse activities such as degradation of contaminants, plant growth promotion, and stress tolerance (1–3).

Soil samples that had been collected from the endemic plant Ulleung-sanmaneul (Allium ulleungense H. J. Choi & N. Friesen), growing in the wild habitat of South Korea, were serially diluted and plated on LB agar. V. paradoxus strain JBCE486 was isolated after 24 h of incubation at 28°C. The genome was sequenced to understand the characteristics of plant growth promotion and salt stress alleviation. Genomic DNA was extracted from a pure culture of JBCE486 that had been cultivated in LB agar at 28°C for 24 h (4). The genome was sequenced by a sequencing company (Macrogen Inc.) using high-throughput sequencing approaches with the Pacific Biosciences (PacBio) Sequel system and the Illumina HiSeq X Ten system. The sequencing libraries were prepared using a PacBio Sequel microbial library construction kit and a TruSeq Nano DNA kit for Illumina following the manufacturers’ protocols. The library insert sizes were 20 kb for PacBio single-molecule real-time (SMRT) sequencing and 350 bp for Illumina sequencing. PacBio long reads were used for *de novo* assembly with the Microbial Assembly application implemented in SMRT Link v8 (PacBio) (5). Pilon was used for error correction with Illumina raw sequence data, with default settings of read length of ≥75 bases and total sequence coverage of >100× (6). PacBio sequencing generated a total of 150,118 subreads (*N*_50_, 10,819 bp), with an average length of 8,336 bp (total, 1,251,527,533 bp). A total of 8,488,730 Illumina paired-end reads were generated, with coverage of 100%. Subreads were mapped against assembled contigs to generate the consensus sequence. Annotation of the genome was performed using the National Center for Biotechnology Information (NCBI) Prokaryotic Genome Annotation Pipeline (PGAP) v2.0 (7). The annotation was validated with Prokka (8) and functionally categorized with the Rapid Annotations using Subsystems Technology (RAST) Server (9). Default parameters were used for all software unless otherwise specified. The genome was composed of a 7,294,742-bp circular chromosome, with a GC content of 66.2%, 6,726 coding sequences (CDSs), 58 tRNAs, and 6 rRNAs. The greatest numbers of genes were involved in amino acid transport and metabolism (647 genes), transcription (554 genes), inorganic ion transport (379 genes), energy production (367 genes), and carbohydrate metabolism (309 genes).

The genome contains genes or gene clusters for the production of indole acetic acid, phenazine, siderophores, and hydrogen cyanide, which are important for plant growth promotion and competition with surrounding microbes. Biosynthetic gene clusters for secondary metabolites were predicted with antiSMASH v6 (10) and confirmed with PRISM v4 (11). JBCE486 was predicted to produce delftibactin A/delftibactin B, colonic acid, bacillobactin, bacteriocins, resorcinol, arylpolyenes, and terpenes.

In view of the versatile metabolism and functional roles of Variovorax paradoxus in plant rhizospheres, the genome sequence will contribute to understanding the mechanisms in the induction of plant stress resistance, which will be valuable for practical use of the strain in the conservation of endemic plants.

### Data availability.

The genome of V. paradoxus strain JBCE486 was made publicly available in GenBank (accession number CP102931). The associated BioProject and BioSample accession numbers are PRJNA869878 and SAMN30330645, respectively. The SRA accession numbers are SRR21599884 and SRR22118462.
